# MYCN as an oncogene in pediatric brain tumors

**DOI:** 10.3389/fonc.2025.1584978

**Published:** 2025-04-29

**Authors:** Adriana Fernandez Garcia, Jayden Jackson, Poorvi Iyer, Elissa G. Oliver, Kosuke Funato

**Affiliations:** ^1^ Center for Molecular Medicine, University of Georgia, Athens, GA, United States; ^2^ Department of Biochemistry and Molecular Biology, University of Georgia, Athens, GA, United States

**Keywords:** MYCN, pediatric brain tumors, tumorigenesis, oncogene, neuro-oncology

## Abstract

MYCN, or N-Myc, is a member of the MYC family of transcription factors, which plays a key role in tumor formation by regulating genes involved in proliferation, differentiation, and apoptosis. MYCN is essential for neural development, especially for the appropriate growth and differentiation of neural progenitor cells, and its aberrant expression contributes to tumorigenesis. Gene amplification and mutations of this gene have been observed in a wide variety of cancer types, particularly in pediatric brain and non-brain tumors, such as neuroblastoma. Previous studies have provided extensive insights into the complex regulatory network of this transcription factor. Additionally, the presence of MYCN alterations in patient tumors serve as a key factor for risk stratification, as it correlates with poorer outcomes, and presents a significant challenge for treatment. Despite its clinical significance, therapeutic targeting of MYCN is challenging due to its structure, nuclear localization, and complex regulatory pathways. Efforts to target MYCN have focused on destabilizing the protein, modulating epigenetic mechanisms, and disrupting its transcriptional network. This review explores the role of MYCN in different subtypes of pediatric brain tumors and highlights novel ongoing therapeutic approaches. However, further research is necessary to develop more effective therapies and improve survival outcomes for patients with MYCN-driven tumor.

## 1 Introduction

**Figure 1:**
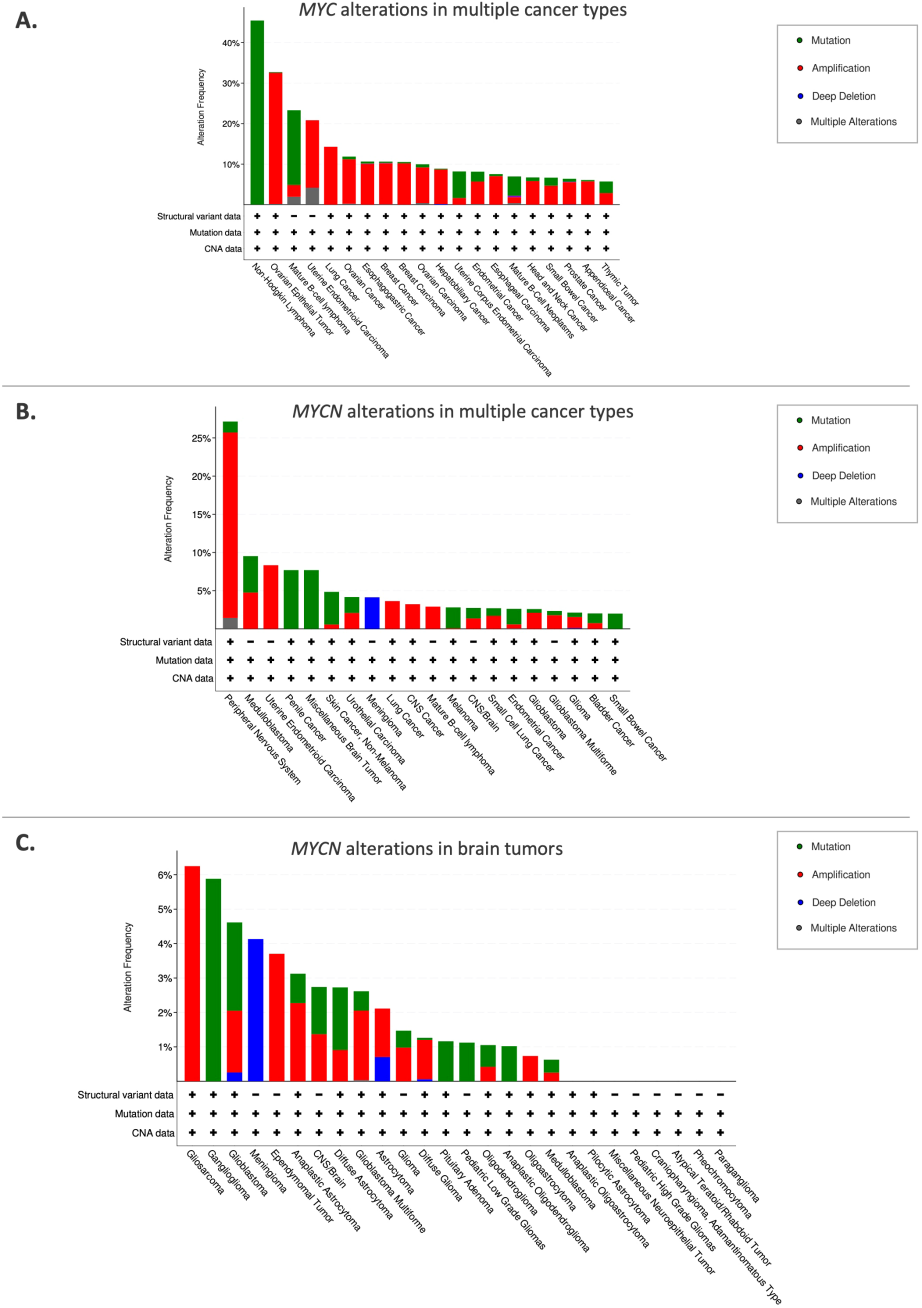
Frequency of MYC/MYCN alterations in various cancer types. **(A)** *MYC* alterations in multiple cancer types. **(B)** *MYCN* alterations in various cancer types. **(C)** *MYCN* alterations in brain tumors. The top 20 cancer types are shown. Sourced from cBioPortal.org.

*MYCN (*or *N-Myc*) is a member of the MYC family of oncogenes, which also includes *C-Myc* and *L-Myc* (1). The *c-Myc* gene (v-Myc myelocytomatosis viral oncogene homolog) was initially discovered in Burkitt lymphoma, a fast-growing type of non-Hodgkin lymphoma (2). MYC family oncogenes act as transcriptional factors, playing a critical role in regulating crucial genes involved in proliferation, differentiation, and apoptosis (3). Overexpression or constitutive activation of a MYC family oncogene can lead to deregulated growth and proliferation, eventually contributing to cancer development. The overexpression of MYC family genes in a wide variety of cancer highlights their role in tumorigenesis (1–5) (**Figure 1A**). Several critical cancer-related pathways, including mitogen-activated protein kinase/extracellular signal-regulated kinase (MAPK/ERK), WNT, transforming growth factor ß (TGFß) and sonic hedgehog (SHH), have *MYC* as a key downstream target (3). Moreover, MYC family proteins are essential for cell cycle regulation and developmental processes such as stemness and cell fate determination (6). For instance, ectopic expression of *MYCN* in neuroblastoma cells can stimulate the re-entry of quiescent cells into the cell cycle and reduce the G1 phase, also decreasing cell attachment to the extracellular matrix (7). On the other hand, when MYCN expression is reduced, cell cycle arrest, differentiation and apoptosis can be observed (7).

Schwab et al. first identified *MYCN*, which was amplified in a panel of neuroblastoma cell lines (4). Albeit a significant functional overlap between MYC and MYCN, their expression patterns are different. While MYC is expressed ubiquitously, MYCN is predominantly expressed in the peripheral and central nervous system (CNS) (8). Aberrant expression of MYCN in the developing nervous system has been linked to the development of neuroblastoma and medulloblastoma, highlighting its role as a potent oncogenic driver (9, 10). In addition to the nervous system, MYCN expression is also present in other tissues, including the reproductive system and urinary system, from which MYCN-altered tumors arise (**Figure 1B**).

*MYCN* is a proto-oncogene that is amplified in various types of cancers, particularly pediatric brain tumors, which are the primary focus of this review (**Figure 1C**). Childhood cancers, especially those that appear very early in life, often exhibit embryonal characteristics (11). Tumorigenic mutations in childhood cancer are more likely to arise in stem or progenitor-like cells, which possess self-renewal properties, and uncontrolled proliferation of these developmentally immature cells results in tumor formation.

**Figure 2:**
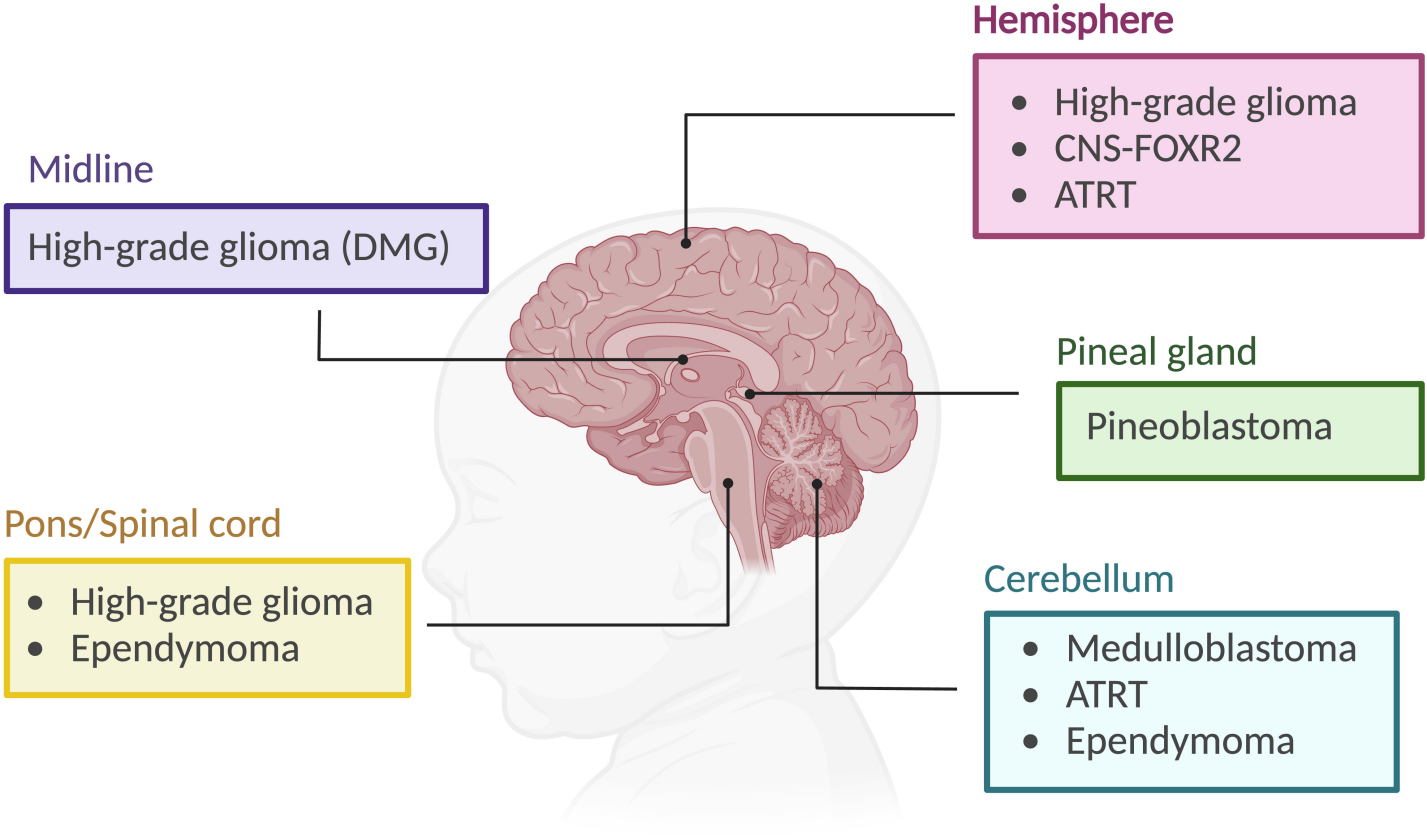
Pediatric brain tumor subtypes and their location. CNS-FOXR2: central nervous system with FOXR2 activation. ATRT, atypical teratoid/rhabdoid tumor. DMG, diffuse midline glioma. Created using BioRender.org.

Pediatric brain tumors are the leading cause of cancer-related deaths in children. CNS tumors are classified based on histological and molecular features, as well as their presumed site of origin within the brain. Embryonal tumors include medulloblastoma, CNS neuroblastoma, pineoblastoma, atypical teratoid/Rhabdoid tumors (ATRT), and embryonal tumor with multilayered rosettes (ETMR) (12). Other subtypes include gliomas, which are categorized by grade and other characteristics, as well as craniopharyngiomas and pineal region tumors (13) (**Figure 2**). MYCN plays a significant role in tumorigenesis, particularly in neuroblastoma, medulloblastoma, high-grade gliomas, and atypical teratoid/rhabdoid tumor as detailed below.

## 2 Overview of MYCN

### 2.1 Structure, function and regulation

The *MYCN* gene, identified in 1983, is one of three members of the MYC family oncogenes (4). These genes are located on different chromosomes and are expressed at specific stages of development (8). Spanning 6,455 nucleotides and consisting of three exons on chromosome 2p24.3, *MYCN* (Gene ID: 4613) encodes a transcription factor (14). Like other MYC family members, MYCN regulates genes involved in the cell cycle, apoptosis, and differentiation (14, 15). MYCN has a short half-life of about 30 minutes, but in MYCN-amplified neuroblastoma cells, MYCN expression can be maintained at a high level (16).

**Figure 3:**
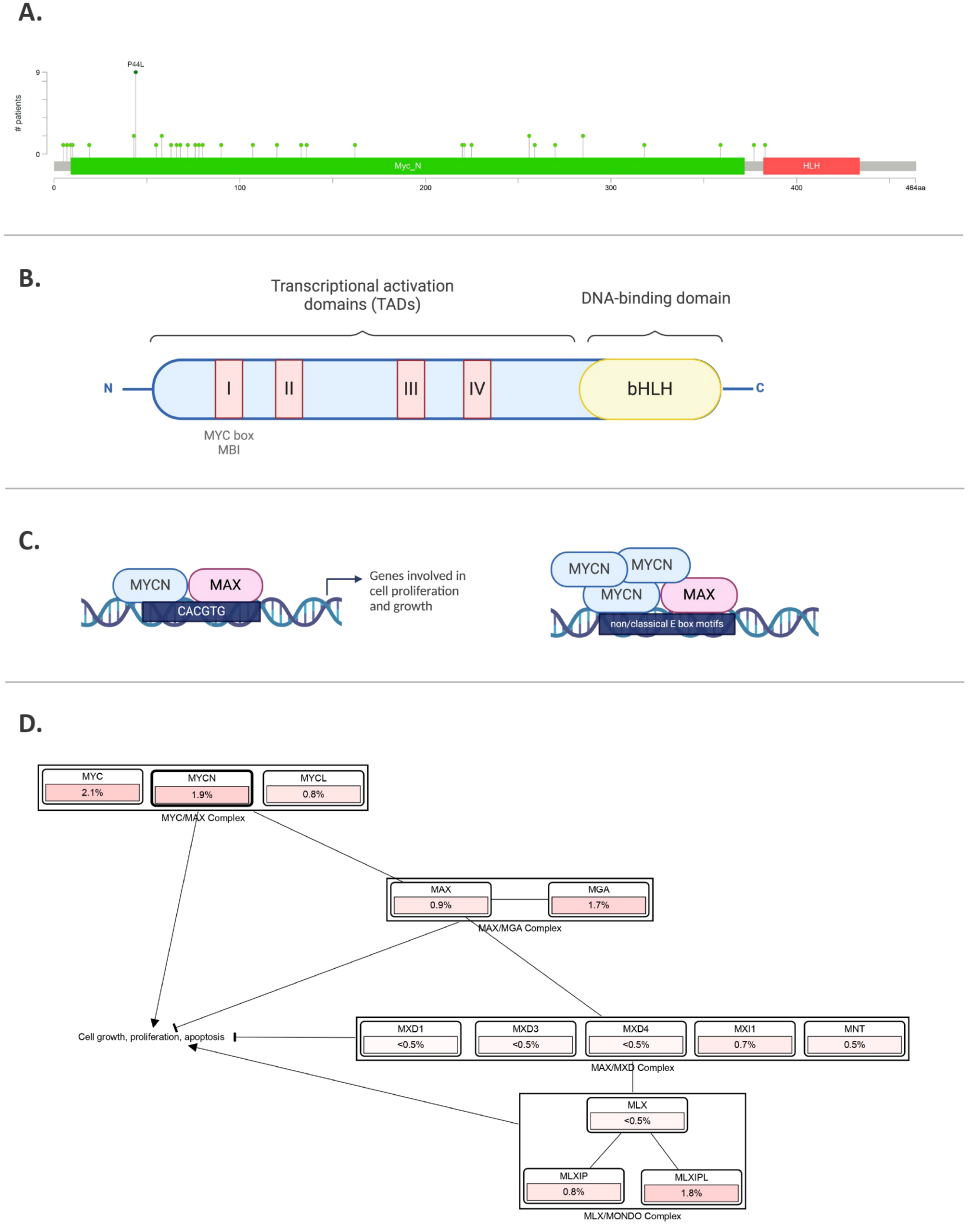
MYCN gene and protein. **(A)** *MYCN* mutations in pediatric brain tumors, featuring hotspots and P44L mutation. Sourced by cBioPortal.org. **(B)** Illustration of MYCN protein structure including different domains. **(C)** MYCN binding to E-box regions in the promoters of target genes involved in cell proliferation and growth. In the right panel, amplified MYCN can bind non-classical E-box sites. **(D)** Pathway analysis, where % represents the percentage of cases in pediatric brain tumors with those alterations. Sourced by cBioPortal.org.

The MYCN protein, with a molecular weight of approximately 63 kDa, comprises 464 amino acids. Similar to MYC, it contains both DNA-binding and transcriptional activation domains (TADs), which include the MYC boxes MB0, MBI, MBII, MBIIIa, MBIIIb, and MBIV, as well as a nuclear localization signal (17) (**Figure 3B**). The DNA-binding domain at the C-terminal contains a basic helix-loop-helix (bHLH) motif that recognizes specific sequences, such as the E-box (CACGTG and CATGTG) (18, 19). In the case of *MYCN* amplification, this specificity is lost, allowing it to bind additional non-classical E-box sites like CAACTG, CATTTG, and CATCTG (19, 20) (**Figure 3C**). Upon binding, histone acetyltransferases (HATs) are recruited to maintain chromatin in a transcriptionally active state (21). The bHLH motif is also essential for protein dimerization with MYC-associated protein X (MAX) (22). All MYC family members form heterodimers with MAX, which preferentially binds to specific target genes (23). MYCN remains functionally inactive until it heterodimerizes with MAX, enabling it to interact with DNA (22). MYC and MYCN share many of the same binding partners, contributing to a more extensive protein network that includes proteins like Max-dimerization partner (MXD), MAX network transcriptional repressor (MNT), and MAX gene-associated protein (MGA) (23, 24) (**Figure 3D**). While MYC/MAX promotes cell proliferation and growth, the MXD/MAX complex functions antagonistically, repressing transcription through histone deacetylases (HDACs). Overexpression of MXD can inhibit MYCN-driven cell proliferation.

Beyond MAX binding, MYCN regulates gene expression through multiple mechanisms. It interacts with WD repeat domain 5 (WDR5), a subunit of the histone H3K4 methyltransferase complex, and can directly regulate target genes by binding to the E-box region of their promoters (25). Interestingly, when MYCN and p53 are highly co-expressed, MYCN can form a complex with tetramerized p53, influencing gene expression through E-box sequences and p53 response elements (26).

**Figure 4:**
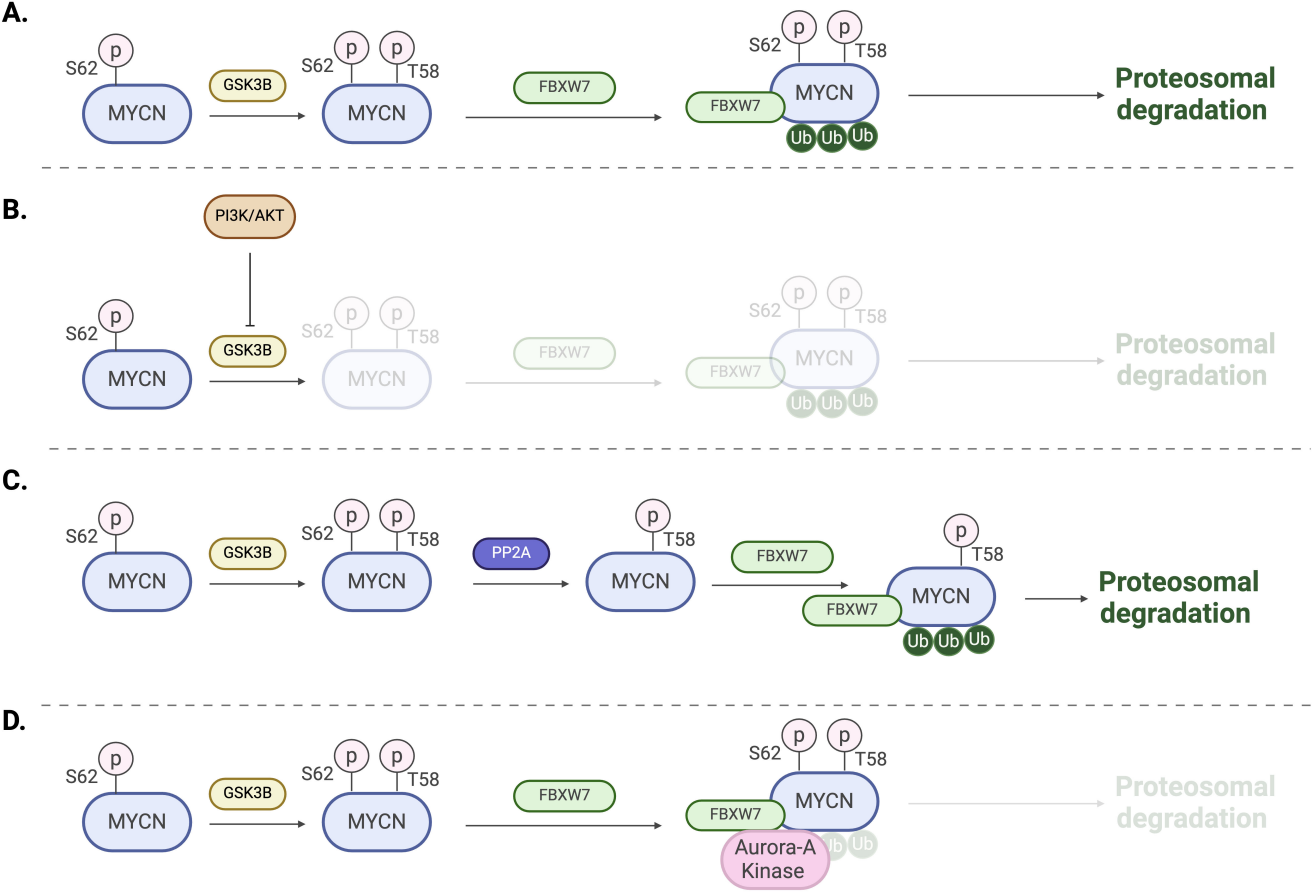
Regulation mechanisms of MYCN. **(A)** Phosphorylation of T58 by GSK3B followed by ubiquitination by the E3 ligase SCF^FBXW7^ and subsequent proteasomal degradation. **(B)** Activation of PI3K and AKT signaling leads to the inhibition of GSK3B and proteasomal degradation. **(C)** Dephosphorylation of S62 by PP2A promotes MYCN ubiquitination and proteasomal degradation. **(D)** Aurora-A binding to MYCN/FBXW7 complex blocking ubiquitination and degradation.

The MYCN protein is regulated in many ways, including phosphorylation. As mentioned earlier, MYC boxes are across the MYCN protein. The MB1 domain plays a crucial role in stabilizing the protein. Phosphorylation of MB1 at serine-62 promotes further phosphorylation at threonine-58 by glycogen synthase kinase 3β (GSK3β), leading to the ubiquitination of MYCN by the E3 ligase SCF^FBXW7^ and its subsequent proteasomal degradation (27, 28) (**Figure 4A**). On the other hand, MYCN stability is also indirectly influenced by the phosphoinositide 3-kinase (PI3K) signaling pathway, which inhibits GSK3β and stabilizes MYCN (29) (**Figure 4B**). Additionally, several other factors, including Protein phosphatase 2A (PP2A), play roles in MYCN protein stability (28) (**Figure 4C**). For example, in MYCN-amplified neuroblastoma cells, Aurora kinase A (AurA) can bind to MYCN and stabilize the protein (30). The catalytic domain of Aurora-A binds to the MYCN/SCF^FBXW7^ complex at flanking sites found on residues 28–89 within MB1 and blocks the ubiquitination mediated by SCF^FBXW7^ (31) (**Figure 4D**).

The N-terminal transcriptional activation domain (TAD), conserved across the MYC family, recruits cofactors that enhance transcription (32). In particular, the MYC boxes facilitate the binding of specific factors; for example, MBI interacts with the positive transcription elongation factor (P-TEFb), which enhances RNA polymerase II elongation, while MBII regulates the binding of the coactivator transcription domain-associated protein (TRRAP), which plays a role in chromatin remodeling (32, 33). Some other cofactors include the acetyltransferase EP300 and components of the super elongation complex, which are essential for the transcriptional activation of MYCN target genes (16, 34). MYCN can also suppress gene expression by interacting with Miz-1 or EZH2 (35–37). Additionally, a study showed that EZH2 promotes MYCN stabilization by counteracting SCF^FBXW7^-mediated MYCN proteasomal degradation (38).

MYCN target genes play critical roles in all cell cycle stages, particularly in the transition from G1 to S phase, which is essential for DNA replication (7, 14, 17). MYCN upregulates genes involved in the cell cycle, such as cyclin-dependent kinase 4 (*CDK4*), minichromosome maintenance (MCM) complex components, Myb-related protein B (*MYBL2*), serine/threonine-protein kinase checkpoint kinase 1 (*CHK1*), inhibitor of DNA binding 2 (*ID2*), and S-phase kinase-associated protein 2 (*SKP2*) (39, 40). This upregulation contributes to increased cell growth and progression through the cell cycle. Notably, inhibition of MYCN has been shown to decrease the G1 to S phase transition rate (41). As described earlier in **Figure 4B**, PI3K promotes MYCN stabilization. It has been shown that the repression of PI3K leads to the degradation of MYCN protein (42). MYCN also negatively regulates p27, which inhibits G1 cyclin-dependent kinases (CDKs) (43).

Furthermore, MYCN plays a dual role in both cell survival and apoptosis. MYCN sensitizes neuroblastoma cells to cytotoxic drugs by cooperatively upregulating BAX (44). On the other hand, MYCN suppresses p53 and its target PUMA (also known as BBC3) by upregulating MDM2 (45). Additionally, a study showed that MYCN upregulates the pro-apoptotic regulator NOXA (20).

### 2.2 Role of MYCN in normal neural development

MYCN plays a critical role in normal brain development, in particular in neural development, by influencing various stages of cellular maturation and neural patterning. Recent studies have shown the close link between neural development and the formation of pediatric brain tumors (46–49). To elucidate the pathogenic mechanisms of MYCN-altered brain tumors and their therapeutic vulnerability, it is crucial to clarify the precise regulation mediated by MYCN during neural development. During early neurogenesis, MYCN expression is critical for the proliferation and maintenance of neural progenitor cells (NPCs) (50, 51). Knockdown of MYCN in mice reduces proliferation of NPCs and results in reduced brain size. MYCN also inhibits the differentiation of embryonic neuronal stem cells, while MYCN knockdown leads to neuronal differentiation. In the developing cerebellum, MYCN promotes the proliferation of granule neuron progenitors (GNPs) by suppressing cyclin-dependent kinase inhibitors (51). Furthermore, studies has shown that deletion or mutation of MYCN can cause Feingold syndrome, a condition defined by reduced brain size and learning disabilities, emphasizing the importance of MYCN expression in maintaining NPCs (52).

In addition to the CNS, MYCN is highly expressed in the developing peripheral neural crest and sympathetic ganglia (53). A series of mouse studies demonstrated that N-myc null mice died between 10.5 and 12.5 of gestation and showed reduced size of the brain as well as cranial and spinal ganglia (53, 54). Given that neuroblastomas originate from neural crest derived precursors, these studies indicate the essential role of MYCN in both peripheral nervus system development and neuroblastoma development. One of the mechanisms through which MYCN exerts its effects is the upregulation of the oncogenic miR-17∼92 microRNA (miRNA) cluster. Specifically, miR-18a and miR-19a inhibit neuronal differentiation by downregulating estrogen receptor alpha (ERα), a nuclear hormone receptor essential for neural differentiation (55).

Given the pivotal role of MYCN in regulating differentiation and proliferation, particularly in normal central nervous system development, dysregulation of MYCN leads to uncontrolled proliferation and impaired differentiation, ultimately driving tumorigenesis. Indeed, a series of mouse studies have demonstrated that MYCN can be an oncogenic driver for glioma, medulloblastoma, primitive neuroectoderm tumor, and neuroblastoma (9, 10, 56).

## 3 MYCN in pediatric brain tumors

### 3.1 Neuroblastoma

While this review focuses on pediatric brain tumors, MYCN also plays a crucial role in neuroblastoma, the most common extra-cranial solid tumor in childhood mainly arising in the adrenal medulla or sympathetic ganglia along the paraventral axis (57, 58). Unfortunately, this pediatric cancer type is responsible for 15% of all pediatric oncology deaths, and MYCN stands out as one of the most critical factors in determining prognosis. Amplification of the *MYCN* locus is found in around 22% of neuroblastomas, and is strongly associated with advanced stages, rapid tumor progression, and poor clinical outcomes. The prevalence of this amplification underscores its significance as a major oncogenic driver in neuroblastoma. Additionally, recent findings have shown alternative mechanisms to increased MYCN protein levels beyond *MYCN* amplification.

Primary neuroblastoma originates from neural crest derived precursors that can differentiate into either sympathetic neurons or chromaffin cells (58). During the development, neural crest cells migrate along a ventral pathway, influenced by signals from somites, ventral neural tube, and other surrounding structures. MYCN expression is elevated in the early stages of development, but decreases progressively, suggesting that appropriate maturation of these cells requires low or absent MYCN expression. Specifically, following migration, MYCN expression is restricted to cells that are actively involved in neuronal differentiation. If MYCN levels remain abnormally elevated beyond the developmental window, these progenitor cells fail to mature and instead proliferate uncontrollably, contributing to neuroblastoma formation. This dysregulation of MYCN expression is associated with the persistence of undifferentiated and proliferative cells, a histopathological feature of neuroblastoma (11, 58). Moreover, histone modifications play a role in regulating MYCN expression. During normal neural crest development, there is a transition from active H3K4me3 mark to repressive H3K27me3 mark, which helps downregulate MYCN during neuronal differentiation. However, in MYCN-amplified neuroblastoma, the H3K4me3 mark persists, sustaining MYCN expression and promoting tumorigenesis (59).

### 3.2 Central nervous system neuroblastoma with FOXR2 activation

CNS neuroblastomas with FOXR2 activation represent a novel pediatric brain tumor subtype identified by Sturm et al. in 2016 (60). This subtype is characterized by the aberrant expression of the transcription factor FOXR2 due to genomic rearrangements (60, 61). CNS-NB FOXR2 is a rare form of neuroblastoma, with a peak incidence at the age of five, typically presenting as a mass in the cerebral hemisphere (61).

A series of studies showed that FOXR2 could bind to both MYC and MYCN proteins, stabilizing them, and activating target gene transcription (5, 62, 63). Since MYCN proteins are considered short-lived and are implicated in tumorigenesis of poorly differentiated tumors, their stabilization by FOXR2 is significant. It is important to note that this post-translational mechanism increases MYCN protein level without *MYCN* amplification. Furthermore, absence of *MYCN* amplification in CNS-NB FOXR2 reinforces the crucial role of FOXR2 in neuroblastoma (63, 64).

### 3.3 Medulloblastoma

Medulloblastoma (MB) is one of the most common malignant brain tumors in children, classified as a WHO grade IV embryonal tumor that arises in the cerebellum. It comprises approximately 60% of all intracranial embryonal tumors, originating from progenitor or neuronal stem cell populations (65, 66). MB exhibits significant clinical and biological heterogeneity, with a peak of diagnosis between 6 to 8 years old, even though it can also occur in infants or adults (66).

Medulloblastoma is divided into four molecular subgroups: WNT-MB, Sonic hedgehog (SHH)-MB, Group 3, and Group 4. The latter two also known as non-WNT/non-SHH (66, 67). Each group displays different prognosis depending on its genetic and molecular profile. Notably, Groups 3 and 4 present the greatest clinical challenges (65). High-level *MYCN* amplification is considered the most recurrent and clinically significant genetic alterations (66, 68, 69).

In the cerebellum development, MYCN have critical functions regulating the proliferation and maturation of precursor cells, therefore playing a fundamental role in orchestrating both normal and abnormal development (67). Its role is especially important during early development, where MYCN functions downstream of SHH, an extracellular signaling molecule essential for growth regulation and differentiation in the developing brain (70, 71). The SHH signaling is the primary driver of the expansion of cerebellar granule cell precursors (GCPs) by directly upregulating MYCN expression (72, 73). This upregulation occurs via a suppression of GSK-3ß, preventing MYCN protein destabilization and subsequent proteasomal degradation (27) (**Figure 4B**). Most of SHH-MB cases are a result of uncontrolled proliferation of GCPs. MYCN expression is strictly regulated during development, with high levels observed in progenitor cells, followed by a downregulation to ensure cell cycle arrest and consequent differentiation and maturation of cells (67).

Among MB subtypes, subgroup SHH-MB is the most well characterized. Patients frequently present gain of chromosome 2, which harbors the MYCN gene, leading *MYCN* amplifications in approximately 7% of cases (66, 74). A significant worse prognosis is observed when patients with *MYCN* amplification also present *TP53* mutations (74, 75). Schwalbe et al. also determined the relevance of clinicobiological heterogeneity within *MYCN* levels, highlighting that SHH-MYCN amplified TP53^mut^ MBs or very high risk MYC-amplified patients exhibited a dismal survival irrespective of treatment. This underscores the urgent need for novel therapeutic approaches (75).

Group 3 MB is characterized by high-level *MYC* amplification, found in approximately 17% of cases (66). The presence or absence of this amplification guides patient stratification, which strongly correlated with prognosis. A unique feature of some tumors from group 3 MB is gene fusions involving MYC and Plasmacytoma Variant Translocation 1 (PVT1), a long non-coding RNA implicated in a variety of human cancers. PVT1 gene fusion is linked to chromothripsis and MYC amplification on chromosome 8q24, further contributing to tumor progression (76).

Beyond *MYCN* amplification in MB, chromosome 17p loss has also been identified as a significant predictor of poor survival. Presence of both is associated with detrimental survival outcomes, especially in patients under three years of age (77). Interestingly, *MYCN* amplification in neuroblastoma has showed association with 17q gain (78).

Further studies have highlighted the role of post-transcriptional regulation in MYC-driven MB cells. Protein arginine methyltransferase 5 (PRMT5) has been shown to interact with MYC/MYCN proteins, protecting them from degradation. Silencing of PRMT5 leads to reduced MYCN levels and impaired tumor cell growth, suggesting that PRMT5 inhibitors could serve as potential therapeutic agents for MYC/MYCN-driven MB (79).

### 3.4 High-grade gliomas

Pediatric high-grade gliomas (pHGGs) are a diverse group of aggressive CNS tumors, accounting approximately 17% of pediatric CNS malignancies (80). While histologically similar to adult glioblastomas, pHGGs are a heterogeneous group that exhibit distinct genetic and epigenetic alterations and molecular characteristics (81). Among the genetic alterations identified in pHGGs, *MYCN* amplification has emerged as a key driver in a subset of highly aggressive tumors. pHGGs can be classified in molecular subgroups based on IDH and histone H3 mutations, including IDH-wildtype, IDH-mutant, H3.3/H3.1 K27-mutant diffuse midline glioma (DMG; also known as diffuse intrinsic pontine glioma or DIPG), and H3.3G34R/V-mutant gliomas (80). The H3.3G34-mutant subgroup, which primarily arises in the cerebral hemisphere, exhibits MYCN upregulation. Specifically, the H3.3 G34V mutation upregulates MYCN expression potentially through histone H3K36me3 mark (82).

The remaining tumors that present neither histone H3 nor IDH1 mutations (H3/IDH1-WT) account for 50% of pHGGs. This has led to the emergence of new molecular subgroups, where MYCN amplifications plays a key role (80). Three molecular subgroups are defined in pontine gliomas: H3K27M-mutant, MYCN-amplified, and MYCN-silent (83). Notably, *MYCN* amplifications are significantly less likely to occur in tumors harboring the H3K27M mutation, and MYCN-amplified tumors maintained the H3K27me3 mark (83, 84). MYCN-amplified pHGGs exhibit distinct clinical and molecular features, including a significant lower median age (9 years) compared to other pHGGs and predominant hemisphere location with a modest predilection for the temporal lobes. Other defining aspects of this subtype are hypermethylation, high-grade histology, and chromothripsis on chromosome 2p that leads to recurrent high-level amplification of *MYCN* and *ID2* (84, 85). Whole-exosome sequencing has revealed a consistent co-amplification of *ID2*, a gene implicated in glioma tumorigenesis, suggesting a potential interplay between MYCN and ID2. It has been suggested that ID2 might acts as a MYCN effector, promoting tumor initiation and maintenance (86). Interestingly, ID2 is also overexpressed in H3K27M-mutant gliomas, suggesting a converging oncogenic pathway among these different subgroups (84).

### 3.5 Atypical teratoid/rhabdoid tumor

Atypical teratoid/rhabdoid tumors (ATRT) are rare and aggressive embryonal tumors of the CNS, which mostly affect infants and young children. These tumors are also characterized by the inactivation of SMARCB1, a tumor suppressor that plays a large role in chromatin remodeling. Molecular studies have demonstrated that ATRTs can be classified into 3 subgroups (87).

Subgroup ATRT-SHH is distinguished by the upregulation of MYCN and key components of the SHH signaling pathway (88). In this subgroup, the loss of SMARCB1 results in enhanced activity of MYCN and SHH pathway genes, which drive tumor cell growth and survival (89). High levels of MYCN also promotes metabolic adaptation, allowing ATRT cells to sustain rapid division and evade apoptotic mechanisms.

The clinical implications of MYCN overexpression in ATRT-SHH remain under investigation. MYCN has been shown to regulate neural progenitor proliferation and differentiation, which may contribute to the neuronal-like transcriptional profile observed in ATRT-SHH tumors (87). Gene enrichment analyses suggest that ATRT-SHH tumors (also referred to as group 1 ATRT) upregulate genes associated with neuronal development and axon guidance compared to other subtypes, suggesting they may originate from a distinct neural precursor (87, 89).

Given its oncogenic role, MYCN presents a potential therapeutic target in ATRT-SHH. Also, the interactions between MYCN and the SHH signaling raises the possibility of combined SHH/MYCN-targeted therapies, similar to those used in SHH-subgroup medulloblastomas (90).

### 3.6 Other childhood brain and non-brain tumors

Pineoblastoma (PB) is a rare, aggressive tumor of the pineal gland. Although there is not much research on *MYCN* amplification in pineoblastoma, studies have shown that MYCN can be highly expressed in this tumor subtype even without gene amplification. Research by Kees et al. established two pineoblastoma cell lines, PER-452 and PER-453, from an 8-month-old patient (91). These cell lines exhibited MYCN expression levels comparable to those in cells with 200-fold *MYCN* amplification, despite lacking actual gene amplification. The study provides an idea that MYCN-driven oncogenesis is not only dependent on gene amplification but can also depend on transcriptional and post-translational regulations. Moreover, these models offer a valuable platform to further explore the molecular mechanisms driving pineoblastoma (91). Further studies could look at the molecular mechanisms behind the enhanced MYCN expression, such as epigenetic modifications, transcription factor involvement, or altered stability of RNAs and proteins. Recent studies have uncovered four PB subgroups based on bulk tumor analysis of DNA methylation and mutation landscapes (92). Interestingly, PB MYC/FOXR2 subgroup was characterized by overexpression of FOXR2, which can bind and stabilize MYCN (62, 63).

Recent studies have identified *MYCN* amplification as a key driver of aggressive spinal ependymomas in pediatric patients (93). An analysis of 13 cases identified a distinct molecular subgroup called spinal ependymoma with *MYCN* amplification (SP-EPN-MYCN), in which all patients exhibited *MYCN* amplification. Despite intensive treatment, SP-EPN-MYCN patients faced significantly poorer outcomes, with a median progression-free survival of 17 months and overall survival of 87 months, showing the aggressive nature of this subtype (93). Additionally, along with these findings, another study reported a 12-year-old male with a spinal ependymoma harboring *MYC* amplification (94). DNA methylation analysis classified the tumor within the SP-EPN-MYCN subgroup, indicating the shared function between MYC and MYCN. These findings underscore the importance of molecular diagnostics in identifying high-risk ependymoma subtypes in pediatric patients. Other pediatric brain tumors with *MYCN* alterations are described in **Table 1**.

**Table 1:** *MYCN* expression, role and clinical relevance in other pediatric non-brain tumors.

Tumor Type	Description	MYCN expression	Clinical relevance
Retinoblastoma (RB)	Rare pediatric eye tumor. MYCN-amplified, RB1-proficient retinoblastomas accounts for 1.5% to 2% of all RB cases (95).	Unlike the typical pathway driven by RB1 mutations, *MYCN* amplification represents an alternative oncogenic mechanism. Overexpression of MYCN drives tumorigenesis. Despite functional *RB1* genes, the retinoblastoma protein (pRb) becomes inactivated due to phosphorylation, leading to uncontrolled cell cycle progression (96).	These tumors exhibit DNA hypomethylation and increased expression of genes involved in protein synthesis, contributing to their aggressive nature (96).
Rhabdomyosarcoma (RMS)	Tumor that affects skeletal muscle and affects children and young adults (97).	MYCN is expressed in both alveolar and embryonal subtypes of RMS, with higher expression levels observed in alveolar RMS and associated with a poorer prognosis (97, 98).	Elevated MYCN expression contributes to tumorigenesis by promoting cell proliferation and inhibiting differentiation. Studies have demonstrated that sustained reduction of MYCN levels in RMS cell lines decreases cell proliferation and increases apoptosis, displaying MYCN as a potential therapeutic target (99, 100).
Wilms tumor (WT)	Fourth most common pediatric tumor, also known as nephroblastoma. A renal cancer found in children younger than five (101).	Analyses have shown that *MYCN* gain occurs in approximately 8.7% to 18.5% of WT cases, with a higher instance in tumors characterized as diffuse anaplasia, a high-risk subtype (102).	This alteration results in poorer relapse-free and overall survival rates (103). MYCN dysregulation in WT can result from various mechanisms, one of which is recurrent somatic mutations like P44L and specific DNA hypomethylation events (104) (**Figure 3A**).
Osteosarcoma	Most common malignant bone tumor, primarily affects children and adolescents. It is known for its aggressive nature and high metastatic potential, particularly to the lungs.	One study by Mukae et al. (2023), MYCN was overexpressed in human-induced pluripotent stem cell (hiPSC)-derived neural crest cells carrying *TP53* mutations, leading to anchorage-independent growth and transformation into highly malignant chondroblastic osteosarcoma. Analysis showed that MYCN-amplified osteosarcoma cells showed amplification of *GLI1*, a gene common in osteosarcoma malignancy.	MYCN knockdown reduced the proliferation of osteosarcoma cells, proving its role as a key oncogenic driver. MYCN-amplified osteosarcoma cases *in vivo* showed worse prognosis (105).

## 4 Clinical implications and targeting MYCN in pediatric brain tumors

Recent efforts to target MYCN in pediatric brain tumors, including neuroblastomas, medulloblastomas, high-grade gliomas, atypical teratoid/rhabdoid tumors, ependymomas, and pineoblastomas, have faced significant challenges. Developing a drug that passes through the blood brain barrier and specifically targets MYCN has been a challenge for researchers due the protein structure, in particular the DNA binding domains which are composed by two alpha helices without apparent surface for binding small molecules (106). More challenges are due to protein’s complex regulatory network, nuclear localization, undefined topology, resistance mechanisms, minimal hydrophobic domains, and intractable effects on healthy transcription; coining the term “undruggable” (107). However, despite the difficulty in targeting MYCN itself, recent therapeutic strategies have emerged focusing on indirect MYCN inhibition by obstructing upstream regulators, repressing transcription, or disrupting downstream interactions using small molecules (108).

### 4.1 Targeting BET

**Figure 5:**
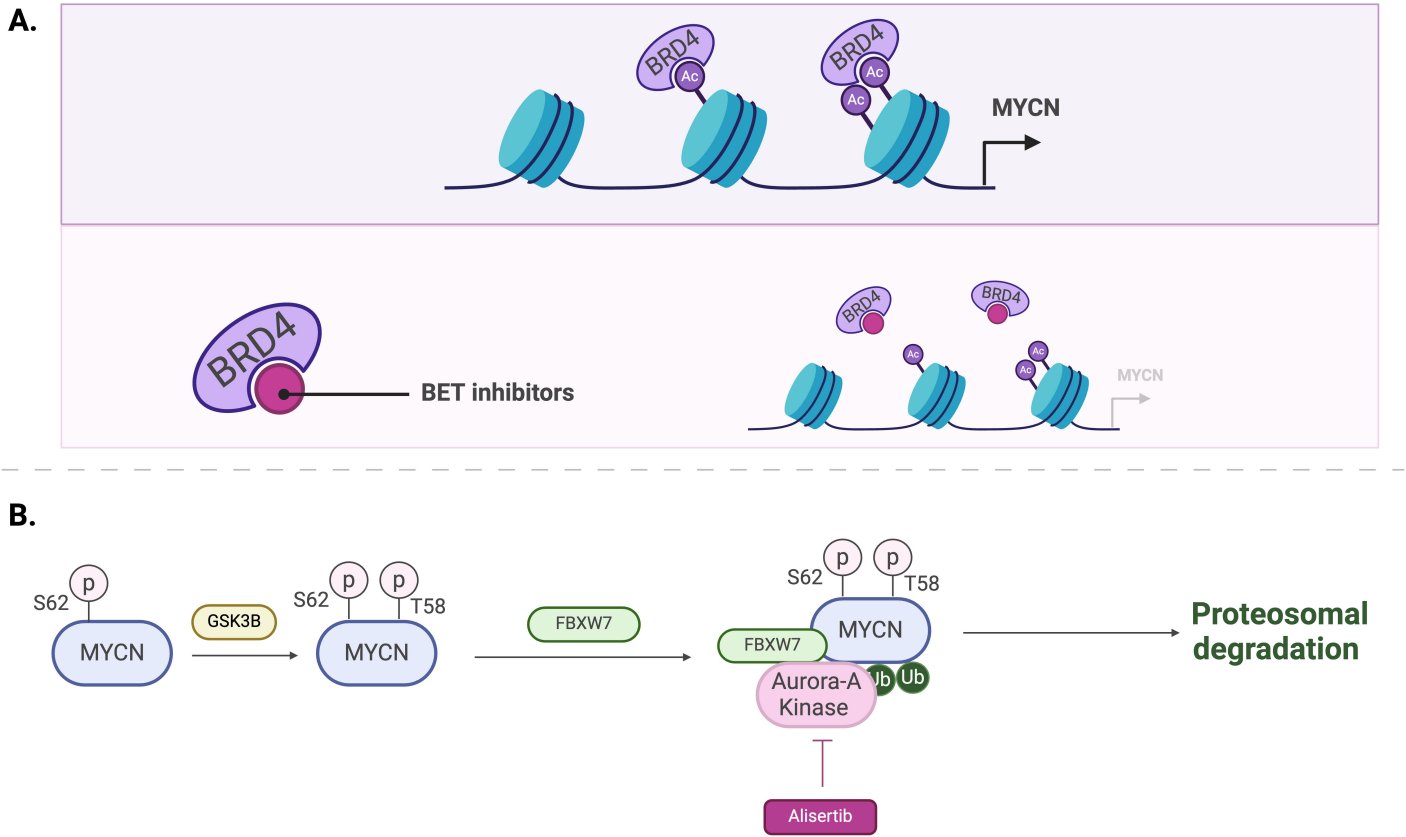
Mechanisms of MYCN regulation by BET inhibitors **(A)** and Aurora-A kinase inhibitors, such as Alisertib **(B)**.

Bromodomain and extra-terminal domain (BET) inhibitors, such as BRD4 inhibitors, have emerged as promising treatment for pediatric tumors by indirectly inhibiting the expression of MYCN. BET proteins are constructed of bromodomains, referred to as BD1 and BD2. These proteins facilitate the transcription of MYCN as well as its target genes by binding to acetylated histones, particularly those located at super enhancers (109) (**Figure 5A**).

BMS-986378, a BRD4 inhibitor, was investigated in a clinical trial which focused on treating pediatric tumors associated with *MYCN* amplification (NCT03936465) (see **Table 2**). The trial encompassed 41 pediatric solid tumor patients associated with *MYCN* amplification, under the age of 21, who exhausted all curable measures prior to the study without any improvement in condition. Phase 1 of the Interventional study began in September 2019 and was completed in March 2024, however, to date, the results have not yet been posted. This trial excluded patients with CNS tumors, leaving the efficacy of BMS-986378 on MYCN-overexpressing pediatric brain tumors unknown.

**Table 2:** Completed interventional clinical trials focusing on targeting regulation pathways of MYCN in pediatric brain tumors.

Identifier number	Study Title	Phase	n	Intervention	Objective	Results
NCT03936465	Study of the Bromodomain (BRD) and Extra-Terminal Domain (BET) Inhibitors BMS-986158 and BMS-986378 in Pediatric Cancer	I	41	BET Inhibitor: BMS-986158 Trotabresib: BMS-986378	Measure MYC protein levels before and after drug use; to investigate the toxicities, response rate, and PK/PD of the drug for pediatric patients; to establish a dosage amount to use in phase 2 of clinical trial	Antitumorigenic properties of BMS-986158
NCT01154816	A Phase II Study of MLN8237, a Selective Aurora A Kinase Inhibitor in Children with Recurrent/Refractory Solid Tumors and Leukemias	II	118	MLN8237: Alisertib	To measure Aurora A Kinase levels before and after drug use; To investigate the toxicities, response rate, and PK/PD of both drugs for pediatric patients; to observe the various of UDP-glucuronosyltransferase gene UGT1A1 when introduced to MLN8237; to study Aurora A Kinase Gene variant- Phe31Ile and Val57Ile	Antitumorigenic properties of MLN8237

Inhibitors are indicated in bold. Information provided from clinicaltrials.gov.

JQ1, another BRD4 inhibitor, is considered a small molecule that inhibits BRD4 binding to the genome and prevents proliferation of cells (110). JQ1 consists of a Triazole ring which allows it to exist within the acetylated lysine binding site using hydrogen bonds (111). Preclinical studies showed that combining JQ1 with Alisertib, an Aurora-A kinase inhibitor, significantly exhibited anti-tumor benefits in neuroblastoma models (108). However, the clinical efficacy of BET inhibitors has been limited by dose-limiting side effects, including grade 3 or 4 thrombocytopenia, fatigue, nausea, vomiting, and diarrhea (112).

MZ1 is a proteolysis-targeting chimera (PROTAC) that was designed based on JQ1 (113). MZ1 binds to BRD4 protein and recruits E3 ubiquitin ligases for degradation (114). Preclinical *in vitro* studies showed that MZ1 reduces the tumor growth rate and, in some cases, induces apoptosis. Additionally, mouse models revealed that MZ1 completely blocks the MYCN downstream pathway as well as the MAPK signaling. MZ1 remains a relatively novel drug with great potential for targeting MYCN (113).

Due to the limitations of single-agent therapies, combination treatments have been investigated for MYCN-amplified tumors. A preclinical study examined the synergistic effects of JQ1 (BRD4 inhibitor) and Alisertib (AURKA inhibitor) in MYCN-amplified neuroblastoma cell lines and mouse models (108). The findings indicated that the JQ1/Alisertib combination enhance survival more effectively than either drug alone (108). While these promising results led to recommendations for further clinical research, clinical trials have yet to be conducted.

BRD4 inhibitors have also been explored in combination with other agents, such as CDK7 inhibitors. Recent studies demonstrated that YKL-5-124, a novel covalent CDK7 inhibitor, combined with JQ1, induced synergistic cytotoxicity *in vitro* and significant tumor regression in patient-derived xenograft neuroblastoma models (115).

### 4.2 Targeting aurora-A Kinase and other MYCN-stabilizing proteins

Inhibiting kinases responsible for the stabilization of MYCN protein stands as a potential therapeutic approach. Aurora-A kinase (AurA) protein is primarily located in the nucleus and is utilized in the M phase of the cell cycle, inducing proliferation of cells. This kinase is also associated with a poor prognosis for patients with neuroblastomas (116). AurA binds to MYCN and prevents its degradation (30, 31). Therefore, inhibiting AurA has been explored as a strategy to reduce MYCN levels. Inhibition of AurA by siRNA knockdown in a *MYCN*-amplified neuroblastoma cell line has shown to reduce MYCN protein levels and lower cell viability (30).

Alisertib (MLN8237) is a synthesized drug that prevents AurA from interacting with MYCN protein (117) (**Figure 5B**). A phase II clinical trial conducted by the Children’s Oncology Group evaluated the potency and effects of Alisertib in relapsed-refractory neuroblastoma pediatric patients (NCT01154816) (118). This trial was performed based on the results from a preclinical study that indicate MLN8237’s ability to inhibit neuroblastoma growth in a mouse model (117). However, although alisertib showed activity in preclinical models and strong pharmacokinetic-pharmacodynamic relationships, objective response rate in children and adolescents from the phase II study was below 5%. Moreover, alisertib treatment led to significant toxicities, with 13% of patients experiencing dose-limiting effects including myelosuppression. The most common grade 3 or 4 toxicities included neutropenia, leukopenia and thrombocytopenia (see **Table 2**).

A recent study demonstrated the combined effect of EZH2, another protein that stabilizes MYCN, and PARP inhibitors in treating MYC-amplified MB. PARP inhibitors, well-known anticancer therapeutic agents, block the PARP mediated repair of single-strand DNA breaks (119). The inhibition of EZH2 significantly enhanced the sensitivity of these cells to PARP inhibitors (120).

### 4.3 Targeting CDK

An alternative strategy to lower MYCN levels involves targeting cyclin-dependent kinase 7 (CDK7) to interfere with the transcription of amplified MYCN in neuroblastoma cells. THZ1, a covalent CDK7 inhibitor, effectively downregulated MYCN expression, resulting in significant tumor regression in a high-risk neuroblastoma mouse model without inducing systemic toxicity or off-target effects. The selectivity of this treatment is due to the preferential downregulation of super-enhancer-associated genes, including MYCN and other oncogenic drivers. These findings suggest that CDK7 inhibition could be a promising therapeutic strategy for MYCN-driven cancers by selectively targeting mechanisms that sustain global transcriptional amplification in tumor cells (115, 121).

## 5 Discussion

The *MYCN* oncogene is essential for neural development. Aberrant expression of MYCN contributes to the tumorigenesis of various embryonal and non-embryonal pediatric brain tumors by regulating important genes involved in neuronal proliferation and differentiation.

Beyond its role in oncogenesis, *MYCN* amplification is a critical factor for patient stratification, with high-risk patients exhibiting higher MYCN levels. It can also serve as a potential prognostic biomarker, contributing to risk assessment and treatment decisions. However, while targeting MYCN remains a significant challenge, emerging therapeutic strategies are being explored. These include MYCN destabilization (Aurora-A kinase inhibitors), epigenetic modulation (BET inhibitors), and global transcriptional amplification (CDK7 inhibitors), all of which show potential in disrupting tumorigenesis.

Significant progress has been made in understanding the role of MYCN in tumorigenesis, although its complex regulatory network, nuclear localization, and other factors have made it a challenging therapeutic target. The development of preclinical models is essential for testing new therapies and exploring drug resistance mechanisms. Future directions should focus on understating vulnerabilities of MYCN-amplified tumors to develop new personalized treatment strategies. Additionally, future research should emphasize on targeting MYCN-regulated pathways and exploring novel combination therapies to enhance clinical outcomes.

Despite efforts have been made to reduce MYCN levels to improve prognosis across multiple cancer types, including pediatric brain tumors, further research is essential to refine therapeutic strategies and enhance survival outcomes in patients with MYCN-amplified tumors.
